# A Rare Case of Homocysteinemia Presenting With Multiple Aneurysms in an Adolescent Boy

**DOI:** 10.7759/cureus.17950

**Published:** 2021-09-13

**Authors:** Ali Hamza, Anum Arif, Ahsin Manzoor Bhatti, Bismah Riaz, Usman Jamil Mughal, Yashfeen Ahmed, Saman Tanveer

**Affiliations:** 1 Internal Medicine, Combined Military Hospital, Lahore, PAK; 2 Vascular Surgery, Combined Military Hospital (CMH) Lahore Medical College and Institute of Dentistry, Lahore, PAK; 3 Internal Medicine, Combined Military Hospital (CMH) Lahore Medical and Dental College, Lahore, PAK; 4 Vascular Surgery, Combined Military Hospital, Lahore, PAK; 5 Medicine, Army Medical College, Rawalpindi, PAK

**Keywords:** atherosclerosis, aorta, aneurysm, pulsatile, thoracoabdominal

## Abstract

Thoracoabdominal aortic aneurysm (TAAA) is primarily a disorder of the elderly; the condition, however, is rare in children, for whom the misdiagnosis is not uncommon. It is one of the leading causes of death in the older age group worldwide, with a 4:1 male to female ratio. There are no real data about the incidence of aortic aneurysms in childhood. Although rare, an aortic aneurysm can be an important cause of mortality in children and adolescents. We present a case of an adolescent boy with a left coronary artery aneurysm, left axillary artery aneurysm, and TAAA type-IV caused by the metabolic disease homocysteinemia. He was referred to our facility when the complicated picture of the disease was discovered.

## Introduction

An aortic aneurysm is a permanent focal dilation of aorta greater than 50% of the adjacent healthy aorta [1]. It most commonly affects the abdominal aorta particularly the infrarenal segment of the aorta. In the older age group, atherosclerosis is the commonest cause of thoracoabdominal aortic aneurysm (TAAA), but in younger patients, there are multiple risk factors for the development of aortic aneurysm including arteritis, connective tissue disorders, congenital, iatrogenic, post-traumatic, mycotic disorders, Ehlers-Danlos syndrome, Marfan syndrome, Takayasu's syndrome, polyarteritis nodosa, and Kawasaki disease [2]. Although rare, homocysteinemia has previously been identified as a risk factor for the development of TAAA [3]. In 2018 in the United States, abdominal aortic aneurysm (AAA) related complications were responsible for 4,903 deaths, with a crude rate of 1.5 deaths per 100,000 [4].

## Case presentation

Our patient's symptoms began five years ago, at the age of 12, as a gradual onset of generalized vague abdominal pain radiating to the back, not associated with meals. Later, he developed undocumented fever along with generalized joint pain for which he was symptomatically treated by multiple pediatricians.

Two years after the onset of initial symptoms, the patient started having palpitations and later developed bilateral pedal edema. His Cardiologist advised a 2D echo which showed a low ejection fraction of <25% along with an aneurysm of the left main stem (LMS) of the left anterior descending (LAD) artery for which he was put on a nonselective B-blocker, loop diuretics, and angiotensin-converting enzyme (ACE) inhibitor and no further workup was advised.

After three years of uneventful period, the patient developed a pulsatile left axillary swelling followed by wrist drop. His CT angiogram was done, which revealed an axillary aneurysm along with a TAAA. He was operated at a local hospital for excision of axillary swelling only, whereas the aortic aneurysm was left untouched due to the high-risk nature of the operation and lack of a Vascular surgeon in the hospital. For the next two years, the patient was referred from one hospital to another in multiple cities before being finally referred to the Vascular surgery department of our institution in May 2021.

The patient was found to be a thin-built boy with a height of 165cm, a weight of 48kg, and a slightly anemic condition upon assessment. There was a 5x5cm non-tender, and non-compressible pulsatile swelling in the left flank. The arterial pulses were normal and the rest of the physical examination was unremarkable. He did not have thin long extremities, no cutaneous hyperelasticity, no joint hypermobility, no arachnodactyly, and no easy bruising of skin was found.

His lab investigations showed low hemoglobin of 10.1g/dL. However, renal function test, liver function test, and hepatitis profile were unremarkable. His serum homocysteine levels were raised to 15µmol/L. Based on these findings, a diagnosis of homocysteinemia was made.

**Figure 1:**
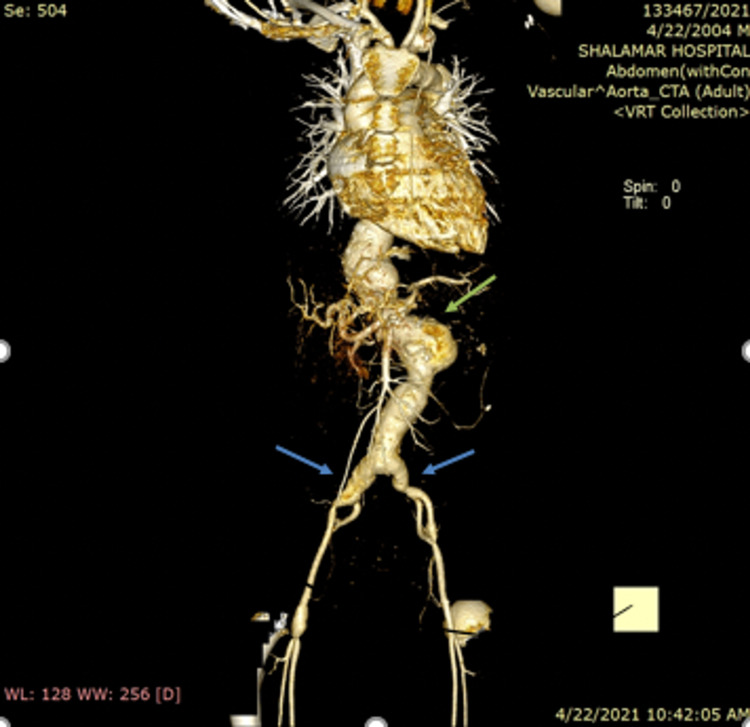
3D constructed view of CT angiogram showing thoracoabdominal aortic aneurysm involving both common iliac veins (blue arrows) and eccentric saccular outpouching of the pararenal part (green arrows)

His CT angiogram revealed a generalized ectatic aorta which became aneurysmal at mid-thoracic level measuring 5x5.5cm as shown in Figure 1.

**Figure 2:**
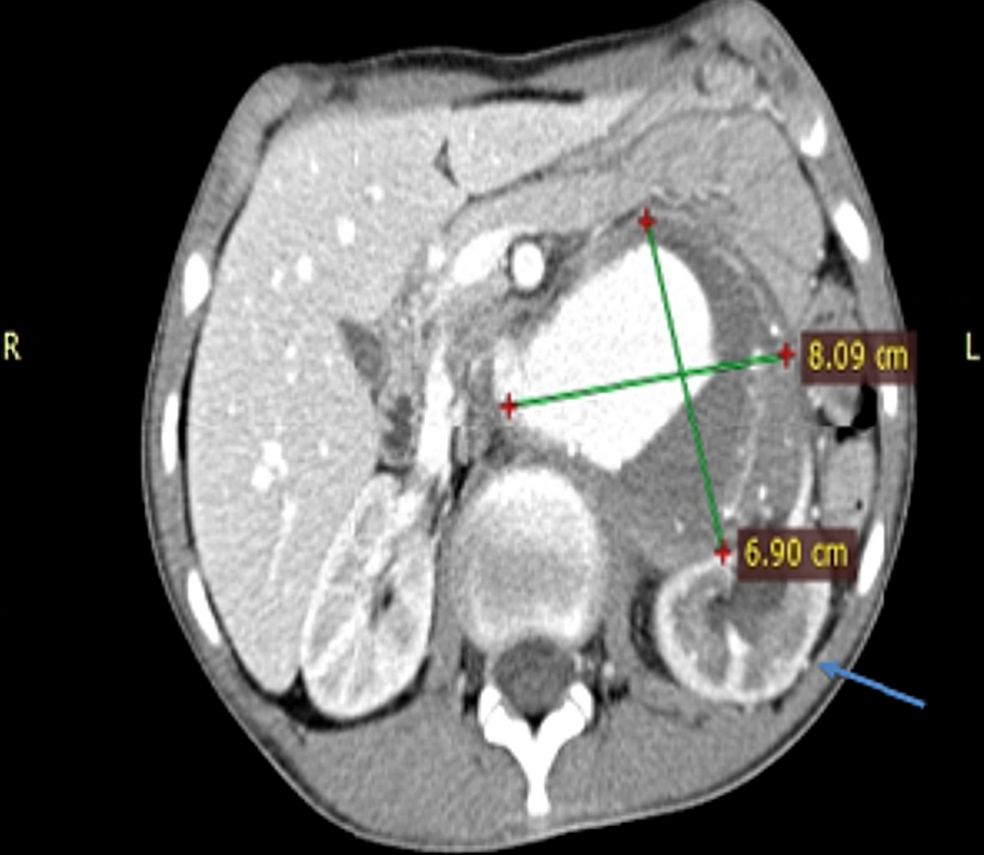
CT angiogram axial view showing the pararenal component of the aneurysm to the largest size. The left renal artery is not visible and the left kidney is also pushed down by the aneurysm (shown by blue arrows)

The largest diameter at the level of renal hila measuring 7cm x 8cm along with markedly thinned out left renal artery as shown in Figure 2.

There was a reduced ejection fraction of 25% on 2D echo and depressed tracer uptake in the anterior septal wall and interventricular septum on MIBI scan.

Understanding the complexity of the clinical picture and the lack of prior experience managing such cases, a multidisciplinary meeting was called in which Vascular Surgeons, Cardiologist, Cardiac Surgeon, and Cardiac Anesthetist were brought on board, and a combined Cardiac and Vascular surgery was planned after a thorough discussion with the patient and his family. Before surgery, high-risk consent was obtained for renal failure, dialysis, paralysis, cardiopulmonary complications, and death.

During the procedure, a midline laparotomy was performed, which extended into a left thoracotomy in the sixth intercostal region, with left medial visceral rotation. The majority of the aneurysm was infrarenal, pressing on the left kidney and straining the left renal vein, which was thinned out, but no left renal artery was detected. Given the disease's intricacy, the left kidney was not mobilized with the rest of the left medial visceral rotation and was held down. After exposing the whole abdominal aorta, arch of aorta, and descending thoracic aorta, it was agreed that just the most aneurysmal section of the TAAA would be replaced with a graft, while the ecstatic arch and proximal descending thoracic aorta would be left intact. A left heart bypass was performed, with a proximal cannula inserted into the left inferior pulmonary vein and a distal cannula inserted into the aortic bifurcation slightly below the anticipated distal clamping point. It is worth mentioning that the patient went into cardiac arrest twice during the surgery, necessitating open-heart CPR and electro cardioversion. As we were performing the first pediatric left heart bypass at our regional center, we modified the pump circuit pressure and speed, and the patient was stable. After stabilization and approval by the anesthetist, the aorta was cross-clamped proximally in the descending thoracic aorta and distally in the infrarenal aorta, 5cm above aortic bi-function. The aneurysm was excised and replaced with a 20mm Dacron tube graft with a proximal end-to-end anastomosis at the bifurcation of the descending thoracic aorta and the abdominal aorta. The celiac artery, superior mesenteric artery, and right renal artery were all anastomosed using the Carell patch technique. We used a sequential clamping approach in which the clamp was shifted proximally after proximal aortic anastomosis to perfuse the spinal vessels, followed by carrel patch anastomosis for visceral perfusion, and finally distal anastomosis. The clamping duration was 40 minutes in total. We did not spend any more time looking for the left renal artery because the aneurysm had been entirely repaired. Transfused were four units of RCC, six units of FFPS, six units of platelets, and six units of cryoprecipitate. Two cheat drains and one abdominal drain were installed. The total skin-to-skin time was 10 hours.

On the third post-operative day, the patient developed left-sided pneumothorax which improved with suction. On the fifth post-operative day, the patient's left lung collapsed which was managed with bronchoscopy and pulmonary lavage. The rest of the postoperative course was uneventful and the patient was discharged on the 12th postoperative day, with referral to a Rheumatologist for management of homocysteinemia and a Nutritionist for dietary modification.

## Discussion

The annual incidence of new AAA diagnosis is approximately 0.67% in the western population affecting predominantly males [5]. Age significantly impacts the incidence, for example, in one study, among males aged 65 to 74 years, the incidence was 55 per 100,000 person-years, increasing to 122 per 100,000 person-years for males aged 75 to 85 years, and further increasing to 298 per 100,000 person-years for those older than 85 [6].

A search of the MEDLINE database found only 41 cases of AAAs in children from 1975 to 2008 [7]. In another study, conducted in 2001, regarding the prevalence of aortic aneurysms among children in which a search from 1966 to 1999 identified 13 reports of aortic aneurysms in children with tuberous sclerosis between 0.5 months and 24 years. The mean age at diagnosis of AAA was 5 years and the mean age at diagnosis of TAAA was 11.7. The youngest patient reported with an aneurysm was 4.5 months old [8]. This demonstrates that aortic aneurysm in children is a distinct disease, and because the number of pediatric cases is limited and dispersed, the origin, natural course, epidemiology, and prognosis are unclear.

Mycotic aneurysm refers to bacterial invasion of the aorta, which remains one of the major risk factors for the development of AAAs in children. An infected aneurysm develops in the setting of bacteremia, direct local invasion of the vessel wall, local spread of septic emboli. In 2013, a case of 15 years old boy was reported with community-acquired methicillin-resistant Staphylococcus aureus (MRSA) sepsis leading to a mycotic aortic aneurysm of the aorta in a previously healthy adolescent with no predisposing cardiac or aortic abnormalities [9]. There was no evidence of infection in our patient; hence, it was not the case.

Multiple cases of aortic aneurysm have been reported in children with connective tissue disorders like Ehlers-Danlos Syndrome (EDS) and Marfan syndrome (MS). Twenty-five percent of patients suffering from vascular EDS (vEDS) experience a major complication, such as arterial dissection or rupture, by age 20. vEDS more commonly involves medium-sized arteries but cases of large vessel arteritis have also been reported. AAA complicating MS is rarely reported in the literature. The clinical history and physical examination of our patient seem to exclude the possibility of a connective tissue disorder.

Congenital AAA can be divided into two groups: Type 1 congenital AAA, a generalized disorder of arterial tissue presenting with aneurysms of other areas, and type 2 congenital AAA, a localized defect of the abdominal aorta. A case was reported in 1988 of a 19 years old boy who has diagnosed with type 2 congenital AAA. The patient had a localized aneurysm of the abdominal aorta, which was surgically treated [10]. There is very little literature available on the congenital aortic aneurysm and no criteria for diagnosis of congenital aortic aneurysm is defined making it difficult to diagnose a case of congenital aortic aneurysm.

Elevated levels of homocysteine in plasma have been shown to correlate strongly with coronary artery disease, stroke, thrombosis, and AAA, independent of other risk factors. In a study done in 2004, it was the observation that patients presenting with homocysteinemia, an autosomal recessive disorder leading to raised plasma homocysteine, also displayed premature vascular disorders that drew attention to the possible relationship between vascular disorder and homocysteine [11]. This was further strengthened by an experimental study, in which an intravenous infusion of homocysteine caused endothelial vascular injury and atherosclerosis [12]. Our patient's plasma homocysteine levels were 5µmol/L higher than the upper limit of normal, indicating that it was the likely cause of his TAAA development. This could be supported further by our patient's history of coronary and axillary aneurysms.

Since Pakistan is a developing country with a recent introduction of vascular surgery, we lack specialized vascular surgery centers. Patients are unsure about where to present themselves, and even primary care physicians are uncertain about where to refer such patients. General surgeons are reluctant to operate on them. This is evidenced by the fact that he was referred from one facility to another for five years following his diagnosis. It should also be noted that because the patient lacked the usual symptoms associated with EDS and MS, he was overlooked by several pediatric surgeons. Our patient not only had a complicated TAAA but also coronary artery disease with low ejection fraction and a coronary aneurysm, which increased surgical risk and split opinion among multidisciplinary team members regarding his best management. Resistance to surgical repair was also due to the previously compromised left renal artery, which might result in post-operative hemodialysis as well as the risk of spinal ischemia and paraplegia.

Once a diagnosis of AAA has been made, efforts should be directed to exclude the presence of aneurysms in other areas and the presence of clinical features referable to connective tissue disorders, any history of infection or bacteremia, any history of similar disease in the family, or any underlying metabolic disorder such as homocysteinemia that might complicate to aneurysmal development.

## Conclusions

Aneurysmal disease although rare in young adults should be among the differential diagnosis in patients presenting with abdominal pain or an abdominal mass that is pulsatile. Due to the lack of a national data registry, we are unable to observe the outcomes of similar cases in our country. Nevertheless, by publishing it here, we want to take the initiative and set benchmarks. Early diagnosis and early referral to a Vascular surgeon can greatly improve the outcome of treatment. Where attempts are made to treat the symptomatic aspect of the disease, there must be some method of identifying the underlying etiology to avoid disease transmission. Such difficult instances necessitate interdepartmental coordination because teamwork is at the heart of outstanding success.
